# Maternal Vitamin D Status and Offspring Bone Fractures: Prospective Study over Two Decades in Aarhus City, Denmark

**DOI:** 10.1371/journal.pone.0114334

**Published:** 2014-12-04

**Authors:** Sesilje Bondo Petersen, Sjurdur Frodi Olsen, Christian Mølgaard, Charlotta Granström, Arieh Cohen, Peter Vestergaard, Marin Strøm

**Affiliations:** 1 Centre for Fetal Programming, Dept. of Epidemiology Research, Statens Serum Institut, Copenhagen, Denmark; 2 Department of Nutrition, Exercise and Sports, Faculty of Science, University of Copenhagen, Copenhagen, Denmark; 3 Clinical Biochemistry and Immunology, Statens Serum Institut, Copenhagen, Denmark; 4 Department of Endocrinology, Aalborg University Hospital, Aalborg, Denmark; 5 Department of Clinical Medicine, Aalborg University, Aalborg, Denmark; Oklahoma State University, United States of America

## Abstract

**Background:**

Studies investigating the association between maternal vitamin D status and offspring bone mass measured by dual-energy X-ray absorptiometry (DXA) during childhood have shown conflicting results.

**Purpose:**

We used occurrence of bone fractures up to the age of 18 as a measure reflecting offspring bone mass and related that to maternal vitamin D status.

**Methods:**

The Danish Fetal Origins 1988 Cohort recruited 965 pregnant women during 1988–89 at their 30^th^ gestation week antenatal midwife visit. A blood sample was drawn and serum was stored, which later was analyzed for the concentration of 25-hydroxyvitamin D (25(OH)D) by the liquid chromatography coupled with a tandem mass spectrometric method (LC-MS/MS). Outcome was diagnosis of first time bone fractures extracted from the Danish National Patient Register.

**Results:**

Vitamin D status was available for 850 women. The median (5^th^–95^th^ percentile) 25(OH)D was 76.2 (23.0–152.1) nmol/l. During follow up 294 children were registered with at least one bone fracture diagnosis. Multivariable Cox regression models using age as the underlying time scale indicated no overall association between maternal vitamin D status and first time bone fractures. However, there was a significantly increased hazard ratio (HR) during childhood for those who had maternal blood drawn in Dec/Jan/Feb compared with Jun/Jul/Aug (HR: 1.75, 95%CI: 1.11–2.74). Adjustment for vitamin D status strengthened this association (1.82, 1.12–2.97), which indicated a potential seasonal impact on offspring fractures independent of maternal vitamin D status. In a sensitivity analysis we found a borderline significant inverse association between continuous concentrations of 25(OH)D and offspring forearm fractures (P = 0.054).

**Conclusion:**

Overall, our results did not substantiate an association between maternal vitamin D status and offspring bone fractures. Further studies on this subject are needed, but the study populations must be large enough to allow for subdivision of fractures.

## Introduction

Adult bone mass may be influenced by factors operating as early as in fetal life [1], and there has in particular been interest on the role of maternal vitamin D status in pregnancy. During pregnancy vitamin D is transported from the mother to the fetus through the placenta in the form of 25-hydroxyvitamin D (25(OH)D), and at birth the concentration in umbilical cord blood is around 80% of maternal blood concentration [2]. Consequently, offspring of mothers with low status of vitamin D may be in a deficient state already from the first days of life. Vitamin D plays a major role in the regulation of calcium homeostasis [3], [4], and studies have found that children born to women with low vitamin D status had lower birth weight, birth length, and lower bone mineral content (BMC) at birth [2].

Different lines of evidence also suggest that maternal vitamin D status may impact offspring bone health in the short as well as the long term, but the studies have shown conflicting results [5]–[9]. As described in several letters in the Lancet during autumn 2013, there is significant disagreement regarding the interpretation of the conflicting results [10]. Accordingly, further studies are needed which address the complicating aspects with regard to age and timing of exposure.

To our knowledge, no earlier studies have focused on the influence of vitamin D status during pregnancy on incident occurrences of offspring bone fractures during childhood. In Denmark we have the opportunity to ascertain this relationship through registry linkages [11]. Bone fractures are to a certain degree determined by bone mineral density (BMD) and may reflect strength of the bone independently of the underlying accident [12]. In a prospective study we have the opportunity to follow the incidences of bone fractures during childhood and adolescence, which may give a more dynamic and functional measure compared with cross-sectional measures at one single point in time, such as measurements from dual-energy X-ray absorptiometry (DXA) scans. In a prospective cohort with follow up over two decades, we therefore examined whether the risk of bone fractures during the first 18 years of life was associated with the maternal status of vitamin D in pregnancy.

## Methods

### Study population

The Danish Fetal Origins Study 1988 (DaFO88 cohort) includes 965 pregnant women who were recruited between 1988 and 1989. This is 80% of a consecutive sample of 1212 women scheduled to attend the routine 30^th^ week antenatal visit at a midwifery practice that covered a geographically well-defined area of the city of Aarhus in Denmark [13]. A self-administered questionnaire was mailed to the women one week before the scheduled visit. During the midwife visit a 15 min structured face-to-face interview was conducted covering medical history, anthropometry, diet, lifestyle and socio-economic factors. A blood sample was drawn and the women provided consent for themselves and their unborn child for participation and later data linkage to Danish health registers. The protocol was approved by the Science-Ethics Committee in Denmark (M-20070157), and the study has been conducted in accordance with the ethical standards in the Declaration of Helsinki.

### Exposure assessment

The blood sample taken in week 30 of gestation was immediately separated into serum, plasma and erythrocytes and frozen at −20°C. Approximately 20 years later, the serum samples were analyzed for concentrations of vitamin D by the liquid chromatography coupled with a tandem mass spectrometric (LC-MS/MS) method (“MS/MS vitamin D” kit from Perkin Elmer, Waltham MA), which is considered to be the most accurate measure of vitamin D status [14]. The stability of vitamin D is considered to be relatively high and unaffected by time of storage [15], [16].

Briefly, 30 uL of serum samples were deproteinized in microtiter plates using 120 uL acetonilrile containing ^2^H_3_-25(OH)D2 and ^2^H_3_-25(OH)D3 as internal standards. The supernatant was transferred to fresh plates and dried under a gentle flow of nitrogen. Subsequently, the samples were derivatized using PTAD dissolved in acetonitrile. The derivitization reaction was quenched with quench solution and the samples were subjected to LC-MS/MS analysis. The LC-MS/MS system consisted of a CTC PAL autosampler (CTC Analytics, Zwingen, Switzerland), a Thermo surveyor LC pump and a Thermo TSQ Ultra triple quadrupole mass spectrometer (Thermo Scientific Waltham, MA). Separation was achieved using a Thermo Gold C18 column (50×2.1 mm, 3 u). The following transitions were used: 619.3/298·1 and 607.3/298.1 for 25(OH)D2 and for 25(OH)D3 respectively, 622.3/301.1 and 610.3/298.1 for internal standards of for 25(OH)D2 and for 25(OH)D3 respectively, and 625.3/298.1 and 613.3/298.1 for the calibration standards of for 25(OH)D2 and for 25(OH)D3 respectively.

We defined the total maternal vitamin D status as the sum of measured 25(OH)D2 and 25(OH)D3 concentrations. Values below the detection limit on 5.9 nmol/l were recalculated as ½ x limit detected for D3 and 0 for D2. For our exposure measure we classified the women according to their concentration of total vitamin D: deficiency <25 nmol/l, insufficiency = 25–49.9 nmol/l (deficiency and insufficiency were classified together), sufficiency = 50–74.9 nmol/l, optimal level 75–125 nmol/l, and high level >125 nmol/l. Our definitions are in accordance with the guidelines from the Danish National Board of Health [17] and the American Institute of Medicine [18]. One outlier was excluded because of a 25(OH)D3 concentration on 367 nmol/l. We defined season of blood draw on the basis of ultraviolet B (UVB) index values from the Danish Metrological Institute: Mar/Apr/Maj, Jun/Jul/Aug, Sep/Oct/Nov and Dec/Jan/Feb.

### Outcome

Outcome measure was defined as first occurrence of any fracture between birth and the date of the 18^th^ birthday. Diagnoses were extracted from the Danish National Patient Register (DNPR) by means of the unique Danish personal identifier (CPR). The DNPR is a nationwide register established in 1977 recording information from all hospital admissions, and from 1995 it also covered outpatient activities and emergency room contacts [19]. The register has nationwide coverage of public hospitals with an almost 100% completeness of recordings and a high precision of diagnoses [11], and in particular for fracture diagnoses [20]. Diagnoses in the register were based on the International Classification of Diseases (ICD). ICD-8 codes were used until 1993 and ICD-10 codes from 1994 onwards. ICD-8 and ICD-10 codes for bone fractures used in this study were respectively: 80009-82999 and DS020-DS029, DS120-DS129, DS220-DS229, DS320-DS329, DS420-DS429, DS520-DS529, DS620-DS629, DS720-DS729, DS820-DS829, DS920-DS929, DT020-DT029. For supplementary analyses, we defined forearm fractures as diagnoses with any of the following codes: 81320, 81321, 81328, 81329 and DS525, DS526, DS525A, DS525B, DS525C. In those cases where the diagnosis is related to an accident, information about the accident is available in the DNPR, including codes for traffic accidents.

### Statistical methods

Cox proportional hazard model was used to investigate the association between total vitamin D status in pregnancy and first time bone fracture risk. In addition, we analyzed the association between season of blood draw and offspring bone fractures. We estimated hazard ratios (HRs) and 95% confidence intervals (CIs) using age in days as the underlying time scale, and included the exposure as a categorical (insufficiency, sufficiency, optimal level or high level) or as a continuous variable. Visual inspection of cumulative residual plots did not indicate violations to the assumption of proportional hazards. We followed individuals in the study sample from date of birth until the age of first bone fracture, or until the defined end of follow up which was the 18^th^ birthday of the offspring. Information about offspring emigration or dead during follow up was extracted from the Danish Civil Registration System [21]. We performed sensitivity analyses where we stratified by age at first bone fracture, where childhood was defined as 0 to 10 years of age and adolescence as 11–18 years of age. Furthermore, we analyzed the association between maternal vitamin D status and offspring forearm fractures and performed sensitivity analyses where we excluded all fractures related to traffic accidents.

Information on covariates used in the multiple regression analyses were gathered from the questionnaire and the interview around gestational week 30. Based on accessibility and current knowledge about vitamin D and fracture risk, the following variables were included as covariates: mother’s age (continuous), pre-pregnancy body mass index (BMI) (continuous), smoking in pregnancy (yes, no), parity (nulliparous, 1 child, 2+children) and sex (girl, boy). Missing covariate values for BMI, education and smoking were substituted using multiple imputation (PROC MI) in SAS; for the other covariates we had complete information. All analyses were carried out using SAS statistical software (version 9.4; SAS Institute, Cary, North Carolina).

## Results

Serum samples were available from 88% of the women in the cohort, which in total provided 850 mother and child pairs with a maternal vitamin D status from gestation week 30. No children were censored due to death, but two children emigrated and were censored at date of emigration. The median (5–95 percentile) total vitamin D status among the 850 pregnant women was 76.2 (23.0–152.1) nmol/l. In our study population, 6.3% had vitamin D deficiency (<25 nmol/l), 74.5% reached sufficient status (≥50 nmol/l), 37.8% were at the optimal level (75–125 nmol/l) and 13.4% had high status of vitamin D (>125 nmol/l). No statistical differences in the characteristics were found between women with available measured vitamin D status and women without measured vitamin D status, except for season at blood draw (P = 0.004). Women without a measured vitamin D status were more often in the 30^th^ week of gestation during the winter months (Dec/Jan/Feb).

Background characteristics of the mothers according to vitamin D status are shown in table 1. Total vitamin D status was significantly associated with parity (P = 0.004), with parous women being more likely to be in deficient or insufficient state. Non-smokers and women with a low range pre-pregnancy BMI were more likely to have sufficient levels although these differences were not statistically significant. Maternal vitamin D status was independent of maternal age, occupational status and sex of the child. Season for blood sampling was highly related to the status of vitamin D (P<0.001).

**Table 1:** Background characteristics of participants in the DaFO88 cohort according to offspring bone fracture (yes/no) and maternal vitamin D status (nmol/l) around gestational week 30 (n = 850) in Aarhus city, Denmark.

		FRACTURE			VITAMIN D STATUS (%)				
	Missing %	Yes n = 294	No n = 556	*p* value	<50 n = 217	50–74.9 n = 199	75–125 n = 321	>125 n = 113	*p* value
MATERNALAGE (MEAN)		29.1	29.0	0.72^a^	28.8	29.3	29.1	28.9	0.62^a^
BMI (MEAN)	3.6	21.3	21.5	0.26^a^	21.9	21.2	21.4	21.1	0.10^a^
EDUCATIONSTATUS (%)									
Unskilled		14.0	12.7	0.85^b^	17.1	10.7	12.3	13.0	0.59^b^
Skilled		25.4	26.4		24.6	27.3	24.9	29.6	
University		39.0	36.4		35.8	35.3	37.9	41.7	
Higheracademic		14.3	16.9		14.4	19.8	16.7	10.2	
Other		7.3	7.5		8.0	6.9	8.2	5.6	
SMOKING									
Never (%)	5.3	57.4	61.2	0.30^b^	55.8	56.0	62.5	67.0	0.12^b^
PARITY (%)									
Nulliparous		58.8	57.0	0.26^b^	46.1	57.8	64.2	61.1	0.004^b^
1 child		28.9	33.4		40.5	33.2	25.9	30.1	
2+children		12.2	9.5		13.4	9.0	10.0	8.8	
SEX, BOY (%)		57.1	49.8	0.04^b^	48.4	55.8	51.7	55.7	0.41^b^
SEASON OF BLOOD DRAW (%)									
Mar/Apr/May		14.0	11.9	0.26^b^	12.4	14.6	16.2	4.4	<0.001^b^
Jun/Jul/Aug		30.8	25.8		8.8	18.1	34.3	72.6	
Sep/Oct/Nov		34.2	37.4		52.1	35.7	29.9	17.7	
Dec/Jan/Feb		21.0	24.8		26.7	31.7	19.6	5.3	

a Two-sided ANOVA for measure of association.

b Two-sided P value from chi-square test for measure of association.

In our data 294 children were registered with at least one bone fracture of which 101 (34%) were located in the elbow and forearm, 74 (25%) in the hand, wrist or fingers, 36 (12%) in the lower leg or ankle, 32 (11%) in the shoulder or upper arm, 30 (10%) in the feet or toes, 19 (7%) in the skull or face, and 2 (1%) in other areas of the body. The overall incidence of first time bone fracture in the cohort was overall 222 per 10^4^ (95%CI: 198–248), for boys 243 per 10^4^ (95%CI: 209–283) and for girls 198 per 10^4^ (95%CI: 166–236).

In our data there was no overall association between total vitamin D status and first time bone fracture (table 2). Adjustment for potential confounders did not affect the estimates considerably, and stratifying by sex did not reveal any sex specific differences in the estimates. In contrast, season of maternal blood sampling was associated with offspring bone fractures in our data (table 3). If the blood sample was drawn in Dec/Jan/Feb, the offspring had a significantly higher HR for fractures (HR = 1.39, 95%CI: 1.01–1.92) compared with Jun/Jul/Aug (reference). Stratifying by age revealed that this was mainly attributed for fractures occurring during childhood until age 10 years. Adjustment for status of vitamin D did not attenuate the association (HR = 1.82, 95%CI: 1.12–2.97).

**Table 2:** The association (hazard ratios and 95% confidence interval) between maternal vitamin D status in gestation week 30 and offspring bone fractures in childhood and adolescence among 850 mother and child pairs from the DaFO88 cohort in Aarhus city, Denmark.

			CRUDE	ADJUSTED*
	n	Cases	HR (95% CI)	HR (95% CI)
ALL AGES, 0–18 Y				
<50 nmol/l)	217	78	1.13 (0.84–1.51)	1.17 (0.87–1.57)
50–75 nmol/l)	199	74	1.20 (0.89–1.62)	1.19 (0.88–1.61)
≥75–125 nmol/l)	321	103	1.00	1.00
>125 nmol/l)	113	39	1.09 (0.75–1.58)	1.10 (0.76–1.59)
*P*-value^a^			0.32	0.27
CHILDHOOD, 0–10 Y				
<50 nmol/l)	217	39	1.04 (0.69–1.57)	1.07 (0.70–1.62)
50–75 nmol/l)	199	42	1.25 (0.84–1.87)	1.26 (0.84–1.89)
≥75–125 nmol/l)	321	55	1.00	1.00
>125 nmol/l)	113	20	1.06 (0.64–1.78)	1.09 (0.65–1.91)
*P*-value^a^			0.59	0.53
ADOLESCENCE, 11–18 Y				
<50 nmol/l)	178	39	1.23 (0.81–1.88)	1.29 (0.84–1.99)
50–75 nmol/l)	157	32	1.13 (0.72–1.77)	1.11 (0.71–1.74)
≥75–125 nmol/l)	266	48	1.00	1.00
>125 nmol/l)	93	19	1.12 (0.66–1.91)	1.13 (0.66–1.93)
*P*-value^a^			0.37	0.35

*Adjusted for maternal age, pre-pregnancy BMI, smoking, parity and sex.

a Continuous values of vitamin D status in the Cox regression model.

**Table 3:** The association (hazard ratios and 95% confidence interval) between season in gestation week 30 and offspring bone fractures in childhood and adolescence among 850 mother and child pairs from the DaFO88 cohort in Aarhus city, Denmark.

			ADJUSTED*	+ VITAMIN D¤
	n	Cases	HR (95% CI)	HR (95% CI)
ALL AGES, 0–18 Y				
Mar/Apr/May	113	35	1.06 (0.71–1.59)	1.05 (0.69–1.59)
Jun/Jul/Aug	247	76	1.00	1.00
Sep/Oct/Nov	300	110	1.26 (0.94–1.69)	1.24 (0.90–1.71)
Dec/Jan/Feb	190	73	1.39 (1.01–1.92)	1.36 (0.96–1.93)
CHILDHOOD, 0–10 Y				
Mar/Apr/May	113	21	1.37 (0.79–2.37)	1.42 (0.80–2.50)
Jun/Jul/Aug	247	34	1.00	1.00
Sep/Oct/Nov	300	58	1.45 (0.95–2.22)	1.51 (0.95–2.40)
Dec/Jan,/Feb	190	43	1.75 (1.11–2.74)	1.82 (1.12–2.97)
ADOLESCENCE, 11–18 Y				
Mar/Apr/May	92	14	0.82 (0.44–1.50)	0.77 (0.41–1.43)
Jun/Jul/Aug	213	42	1.00	1.00
Sep/Oct/Nov	242	52	1.10 (0.73–1.66)	1.02 (0.65–1.59)
Dec/Jan,/Feb	147	30	1.08 (0.67–1.73)	0.99 (0.60–1.65)

*Adjusted for maternal age, pre-pregnancy BMI, smoking, parity and sex.

¤ Adjusted for maternal age, pre-pregnancy BMI, smoking, parity, sex and maternal vitamin D status in gestational week 30.

In the sensitivity analysis, where we looked specifically at forearm fractures, we found no significant associations and neither when we adjusted for potential confounders (data not shown). However, when entering the exposure as a continuous variable in the analysis there was a borderline significant inverse association in the adjusted analysis (P = 0.054). Exclusion of bone fractures caused by traffic accidents had no effect on the estimates. When season at blood draw was used as exposure we found a significantly higher HR when the blood was drawn in Dec/Jan/Feb compared with Jun/Jul/Aug (HR = 2.47, 95%CI: 1.28–4.79). When we adjusted for maternal status of vitamin D a significantly higher HR for Dec/Jan/Feb persisted (HR = 2.17, 95%CI: 1.07–4.39).

## Discussion

Overall, there was no significant association between maternal vitamin D status in gestation week 30 and offspring bone fractures from birth until 18 years of age in the DaFO88 cohort. However, in sensitivity analyses we found a borderline significant inverse association (P = 0.054) between vitamin D status in pregnancy and offspring forearm fractures. We also found significantly higher rates of fractures during childhood among offspring whose mothers had blood samples drawn during the winter vs. summer. Adjustment for vitamin D status did not attenuate this association as would be expected if the association were due to seasonal variation in vitamin D status, rather it was strengthened upon adjustment. Our finding may be a consequence of residual confounding, but may also be a consequence of selection bias, since 35.5% of the women had the blood drawn during the summer (Jun/Jul/Aug) and only 13.3% during the winter (Dec/Jan/Feb) in the two-year period of data collection. However, our finding of a seasonal effect may also be true, since season of birth has been found to be associated with later bone fracture risk in older women and men aged 65+ [22]. In that study, the authors suggested their findings to be caused by seasonal variation in vitamin D status during pregnancy. However, if our results are reliable, there might be other aspects underlying a seasonal variation during pregnancy on bone fracture risk later in life independently of vitamin D status. Factors such as dietary habits, physical activity, and mental health problems have been shown to be associated with markers for bone health in adults [23]–[25], and most likely, they may also have an impact on fetal bone mineralization during pregnancy [26], [27]. Several of these factors exert seasonal variation [28], [29], and it could be speculated that they might partly explain our findings regarding season of blood sampling and offspring bone fractures.

The present study is to our knowledge the first to investigate whether maternal vitamin D status predicts offspring bone fractures. Other studies have investigated the effect on offspring BMC and BMD measured by DXA: Javaid et al. [5] showed a direct association between maternal vitamin D status during pregnancy and BMC and BMD in 160 offspring at 9 years of age. In a study of 203 neonates, Ioannou et al. [6] were unable to detect any relevant association between maternal vitamin D status and BMC and BMD shortly after birth, although they did indeed find a positive correlation between maternal vitamin D status and fetal femur volume measured by three-dimensional ultrasound. In the ALSPAC cohort Sayers and Tobias [7] found supporting evidence that measures reflecting intensity of UVB radiation during pregnancy were positively related to BMC and BMD in 6995 children of 9.9 years of age. But remarkably, Lawlor et al. [8] in the same cohort found no relevant association between maternal vitamin D status in pregnancy and BMC in 3960 offspring at 9–10 years of age. Furthermore, they showed that the association with UVB radiation found in the first ALSPAC study disappeared when they adjusted for offspring age at the DXA scan. Most recently, Zhu et al. [9], in the Australian Raine study found a lower peak bone mass in 341 offspring at 20 years of age, when the maternal vitamin D status was below 50 nmol/l. The previous studies applied a cross-sectional measure of outcome using DXA scans at one time point, which implies incomparability with our results. Bone fractures are to a certain degree determined by BMD [30], [31], but the surrounding circumstances also influence bone fracture risk. Bone fractures require an accident or impact of great force to come into play, and are thus only partially determined by the level of BMD.

One potential explanation for the null association between maternal vitamin D status and offspring bone fractures may be the low prevalence of vitamin D deficiency in our study population. If a true underlying beneficial effect of maternal vitamin D status on offspring bone health exists and is limited to very low levels, we may not be able to detect such a relationship in our data, where only 6.3% of the study population had a vitamin D status below 25 nmol/l and 25.5% had a status below 50 nmol/l. In contrast, Zhu et al. [9] reported 38.7% in their study population to have a vitamin D status below 50 nmol/l. Consistent with temporal trends, there were also fewer vitamin D deficient mothers in our study sample compared to two later Danish studies, which measured vitamin D in pregnant women [32] and in a general population sample [33] approximately 10 years later.

Another potential explanation for the null association may be the small study sample, which makes further subdivision of fractures impossible. We found a borderline significant inverse association between vitamin D status in pregnancy and offspring forearm fractures. However, the number of forearm fractures in the cohort was relatively low, which prevented stratification by age and sex. Forearm fractures may serve as a relevant and valid indicator of low BMD compared to other types of fractures, since a review from 2010 concluded that there is evidence for an association between BMD and childhood risk of forearm fractures [31], and since this particular type of fracture often occurs as a consequence of falls on the same level and thus to a lesser extent are related to serious accidents. It would be of great interest to conduct similar analysis to investigate the association between maternal status of vitamin D and offspring forearm fractures in a study population large enough to allow such subdivision of fractures and further stratification by vitamin D status.

The strengths of our study includes usage of data from a high quality registry, which has been updated and improved over time [11]. In 1995, the DNPR was extended to include outpatient activity information and emergency room contacts. Since bone fractures mainly are treated in the emergency room, a significant proportion of fractures occurring prior to 1995, are likely not to have been included in our data. Until 1995, we only have access to diagnoses recorded during hospitalization, which may explain why maternal vitamin D status in our data is unassociated with offspring bone fractures.

A major limitation in the study is the lack of information about offspring weight and height during childhood. Weight and height may be related both to maternal vitamin D status and offspring bone mass, and our results may be confounded when those data are missing. Likewise, we do not have information on physical activity level, duration of breast-feeding or diet during childhood. Furthermore, observations have shown that infant vitamin D deficiency can be eliminated as early as 9 months postpartum, if vitamin D drops with 10 µg of vitamin D per day are given as recommended [34]. Thus, we cannot preclude that early vitamin D supplementation impacted offspring bone fracture risk in our data and has blurred an association with maternal vitamin D status. Calcium intake and physical activity level are also essential for bone mineralization during childhood and adolescence [35], and level and type of physical activity influences the risk of bone fractures [36]. In this context, a potential negative impact of maternal vitamin D deficiency may have been blurred in our data, since data are lacking on potentially important confounders. On the other hand, in the recent ALSPAC study [8] it was shown that offspring level of physical activity and dietary vitamin D intake were not associated with maternal vitamin D status [10]. If this also is the case in the DaFO88 cohort, physical activity level and childhood vitamin D intake could did not act as confounders. Thus, including them in our analyses would not have altered the null associations that we observed.

In conclusion, our data indicated no overall association between maternal vitamin D status during pregnancy and offspring bone fractures in a prospective cohort with 18 years of follow-up through high-quality registries. Further studies on this subject are needed, but such studies must be conducted in study populations large enough to allow for investigation of seasonal variation, subdivision of fractures and further stratification of study participants by vitamin D status.
